# Sulfur Dioxide Degradation by Composite Photocatalysts Prepared by Recycled Fine Aggregates and Nanoscale Titanium Dioxide

**DOI:** 10.3390/nano9111533

**Published:** 2019-10-29

**Authors:** Xue-Fei Chen, Shi-Cong Kou

**Affiliations:** 1 School of Materials Science and Engineering, Nanjing University of Science and Technology, Nanjing 210094, China; chenxuefei@email.szu.edu.cn; 2 College of Civil and Transportation Engineering, Shenzhen University, Shenzhen 518000, China

**Keywords:** construction waste, nano-TiO_2_, recycled fine aggregate, SO_2_ degradation, composite photocatalyst, photocatalytic mortar

## Abstract

To alleviate the heavy burden on landfilling, construction and demolition wastes (C&DWs) are recycled and reused as aggregates in cementitious materials. However, the inherent characteristics of recycled fine aggregates (RFA), such as the high crushing index and high-water absorption, magnify the reusing difficulty. Nevertheless, attributing to the high porosity and high level of calcium hydroxides existing in the old mortar, RFA is featured with a high specific surface area and a high alkalinity. These features are useful to augment the total photo-degradation of SO_2_ by nano-TiO_2_ (NT) intermixed mortar, leading RFA to be an excellent potential carrier to load nano-TiO_2_ and prepare the composite photocatalyst. Hence, this study proposed to load NT onto the surface of RFAs and river sands (RSs) (the control) by the soaking method, preparing composite photocatalysts denoted as NT@RFA and NT@RS, respectively. The prepared composite photocatalysts were then utilized as sands in photocatalytic mortar to evaluate for SO_2_ degradation. Experiments identified a 50% higher amount of NT was loaded onto the surface of FRA relative to the control. This higher loading amount plus higher alkalinity ultimately translated into a higher photocatalytic activity. In addition, the mortar containing NT@RFA exhibited 46.3% higher physiochemical absorption and 23.9% higher photocatalytic activity than that containing NT@RS. In addition, the durability, embodied by the reuse and anti-abrasive properties, of NT@RFA exceeded that of NT@RS. The overall findings reveal that the NT@RFA not only garners beneficial effect from the high porosity but also generates positive effect from the high alkalinity. Though a number of studies deal with building materials with NT, this study is the first to load NT onto RFA and prepare composite photocatalysts which were then used as fine aggregates in building materials. Consequently, this study proves the potential high-added-value reusability of RFA in green construction materials and provides a low-cost, high-efficiency approach to degrade atmospheric SO_2_.

## 1. Introduction

Construction and demolition wastes (C&DWs) are considered globally important because billions of tons are generated annually [1,2]. In these wastes, recycled concrete is a dominant component. Because landfilling triggers several environmental problems such as land wasting, water pollution, and air contamination, C&DW is generally collected, crushed, and sieved into aggregates or powders to produce new building materials [3,4,5]. However, such low added value greatly limits the practical use of all produced C&DW. In addition, the produced aggregates can cause manufacturing problems in rheology [6,7], mechanics [8], and durability [9], particularly when incorporated in large proportions, because of their disadvantages of high porosity, high crushing index, and high water adsorption.

To enhance the use value of recycled aggregates, particularly recycled fine aggregates (RFA), photocatalytic technology is considered, expecting to transform C&DWs into functional (photocatalytic) products. The so-called photocatalysis means the separation of photogenerated carriers in semiconductor materials under certain wavelength illumination. The photogenerated electrons and holes combine with ions or molecules to form oxidative or reductive active free radicals, which can degrade organic macromolecules into carbon dioxide or other small molecular organic compounds. Among all semiconductor photocatalysts, zinc oxide (ZnO) is a promising candidate for photo-degradation [10]. Besides, once the surface modification by a flame-based method is applied to ZnO, ZnO nano- and microwires can be synthesized. This surface modification caused by the grown ZnO nano- and microwires is reported to endow ZnO with an augmented decomposition rate for methylene blue [11]. In addition, the tetrapod-shaped ZnO (T-ZnO) is also verified to rely on the porous networks to accommodate cellular materials such as carbon nano-onion (CNO), and to further ascend the photocatalytic efficiency [10]. Specifically, a hybrid composite based on T-ZnO and carbon nano-onion (CNO) is prepared based on a facile one-step process that has an easy accessibility of the characteristic features of both T-ZnO and CNO. The joint effect ultimately translated into a >90% photo-degradation of 2,4-dintrophenol under visible light within 140 min [12]. This research highlights the feasibility of combining photocatalysts with other materials and making composite photocatalysts with augmented photocatalysis. Apart from ZnO, nano-TiO_2_ (NT) is also a commonly used photocatalyst, particularly in the construction industry.

Traditionally, nanoscale TiO_2_ is directly mixed with cement, water, and aggregates (natural aggregates or recycled aggregates) to produce photocatalytic mortar with air purifying effect, antibacterial effect, and self-cleaning effect. Interestingly, the high porosity, a key feature of RFA, is proved to boost photocatalysis since a higher porosity leads to a corresponding higher sorptivity of pollutant molecules [13]. In addition, the high alkalinity, another key property of RFA because of the calcium hydroxides present in the old mortar of RFA [14], is verified to augment the total degradation of acidic pollutant gases such as sulfur dioxides. Previous studies revealed that high alkalinity benefits the photocatalytic degradation of acid pollutant gases such as NO_x_ [15,16] and SO_2_ [17] because acidic gases are attracted and adsorbed by the alkaline constituents, namely portlandites, of hydrated cement paste. This increases the potential for those pollutants to react with HO_x_ radicals deposited at the cement–TiO_2_ interface of the composite [15]. Some acid gases are directly degraded by physical or chemical sorption in the presence of alkaline materials. Actually, it is observed that the silicate coating with alkaline nature can induce physi-absorption and chemi-absorption, which is similar to neutralization, and thus contributes a ~10% higher sulfur dioxides removal relative to epoxy that is not alkaline [17]. Overall, the presence of RFA is beneficial to promote the degradation of sulfur dioxides.

For the widespread use of photocatalytic technology in construction industry, previous researchers combined NT with cementitious materials to develop functional photocatalytic products by methods including intermixing [13], dip-coating [18], and spray-coating [18]. Particularly, Faraldos et al. [18] once conducted a pioneering work to spray and dip-coat an effective photocatalytic coating onto the surface of concrete materials. The coating, even when thin and diluted, was proved to hold extreme excellent photocatalytic performance over NO (conversion rate was even higher than 90%) and over methylene blue dye. Photocatalysts, including NT, have two main drawbacks of high use cost and poor dispersion performance. The traditional intermixing method has to use large quantities (usually 3–5% by weight of cement or even 10%) of NT particles to generate measurable photocatalytic efficiency, which inevitably triggers the problem of high cost but low efficiency, as mentioned above. To solve the problem and fully take advantage of characteristics of RFA, we attempted to load NT onto the surfaces of recycled fine aggregates by the soaking method and prepare composite photocatalysts (CP). The extremely low cost (almost zero) of RFA reduces the use cost of photocatalysts and the carrier enlarges the photocatalyst particle size, thereby boosting the dispersibility of the CPs in cementitious materials.

The high porosity of recycled aggregates contributes to the absorption of NT solution. By the soaking method, the NT particles coat the surface and accumulate in the pores by physical and chemical bonding [19]. In our previous research [20], CPs prepared using recycled clay brick sands and NT showed excellent NOx and methyl orange removal with the NT concentration of 1 g NT per 100 mL water. Their improved performance relative to river sand-based CPs indicated the benefits of the high porosity. These advantages were attributed to the high holding capacities of the final products and their high surface areas, which permitted the effective irradiation of more nanoparticles by light.

Hence, the efficacy of the lower-cost, higher-efficiency prepared RFA-based CPs was investigated. This research proposed a feasible application of photocatalytic technology to boost the reuse value of recycled construction wastes. The high porosity and alkalinity of the RFA were expected to promote the photocatalytic efficiency of CPs relative to that of NT alone. This study analyzed the mechanical performance of mortar incorporating RFA-based CPs, the prepared CP characteristics, and the durability of the RFA-based CPs, as measured by anti-abrasion and reutilization capability. In particular, the entire degradation process of SO_2_ was also studied. Overall, the research combined RFA and NT into a composite photocatalyst (NT@RFA) and described a theoretical functionalization of a common construction materials (mortar) incorporating with NT@RFA as a photocatalytic mortar for atmospheric pollution degradation. The novelty of this study is the first combination of nanoscale photocatalyst (NT) with recycled building materials. The study contributes the characterization of NT-functionalized RFA-based mortar as a photocatalytic material for atmospheric SO_2_ degradation.

## 2. Materials and Methods

### 2.1. Materials

ASTM Type 1 Portland cement was used as the cementitious material. The recycled fine aggregate (RFA) was not directly sourced from C&DW; instead, they were fabricated in the laboratory by crushing and sieving old concrete (water–cement ratio = 0.5, cured for 90 days) to ensure homogeneity. The water–cement (w/c) ratio was kept 0.5 because this w/c ratio lead to a less compacted concrete relative to the w/c ratio of 0.35 or 0.25. That is, the porosity of the prepared concrete was relatively large, which was a key factor in this study. The concrete was cured for 90 days before crushing because it was used to simulate the actual recycled concrete. That is, the concrete after 90 days curing was assumed to be equivalent to recycled concrete since the cement hydration process was largely completed within the first 90 days. In particular, the recycled fine aggregates used in this study were sourced from the concrete prepared in lab rather than from an actual demolition site because the former relative to the latter had better homogeneity, which was crucial for the repeatability of the research. River sands (RS) were common river sands provided by a local building material company. All sands including RFA and RS had a single gradation of particle size of 1.18–2.36 mm. The physical and chemical properties of these materials are given in Table 1. The photocatalyst was Degussa P25, a nanoscale TiO_2_ particles (20–50 nm) comprising 75% anatase and 25% rutile.

### 2.2. Sample Preparation

The RFA based CPs (NT@RFA) and RS based CPs (NT@RS) were prepared as per the mix proportion shown in Table 2. 

**Figure 1:**
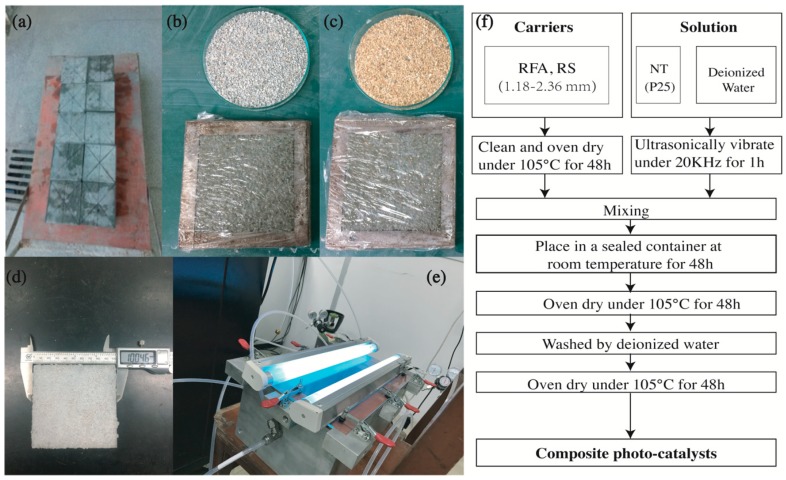
Experimental photos: (**a**) Recycled concrete prepared in the laboratory; (**b**) Recycled fine aggregate (RFA) group; (**c**) River sand (RS) group; (**d**) final specimen containing NT@RFA; (**e**) photocatalytic reactor; and (**f**) Composite photocatalyst (CP) preparation flowchart.

**Figure 2:**
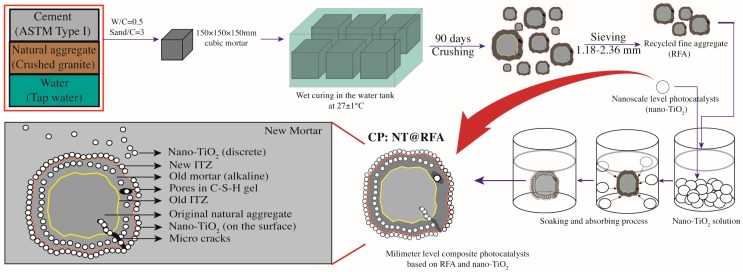
Schematic diagram of the preparation procedures of NT@FRA and the structure of final NT@FRA.

The specific preparation procedures and final product are illustrated in Figure 1, while a schematic diagram is available as Figure 2. Specifically, each 80 g RFA (single gradation of 1.18–2.36 mm) in the clean and oven dry status was soaked in the NT solution (pre-ultrasonic vibrated under 20 KHz for 1 h) with the concentration of 1 g NT per 100 mL deionized water. Then, the obtained mixture was placed in a sealed container for 48 h to make NT fully absorbed in pores of RFA and loaded on the surface of RFA. Next, the composites were oven dried for another 48 h. Afterwards, the dried samples were cleaned by deionized water and again oven dried for 48 h to obtain the final product, namely NT@RFA. The counterpart of NT@RS, which was produced from RS and NT following the same procedures, was also prepared for reference. In particular, both the RFA and RS were ultrasonically cleaned by deionized water and then oven dried before soaking into the NT solution, expecting to remove the impacts generated by impurities. The NT@RFA was prepared by the soaking method that is expected to form a kind of physical bonding caused by physical adsorption, also known as van der Waals adsorption. It is caused by the interaction between adsorbate and adsorbent molecules, which is also called van der Waals force. Since van der Waals force exists between any two molecules, physical adsorption can occur on any solid surface. It is the theoretical background of the preparation procedure of composite photocatalysts.

The CPs were then used as sands to prepare mortar specimens with the constant sand ratio of 2.5 and w/c of 0.5 as per the mix proportion shown in Table 3. In particular, two types of specimens were prepared. One was a cubic specimen in dimensions of 40 mm × 40 mm × 40 mm, using for compressive strength testing; the other one was a board specimen in dimensions of 100 mm × 100 mm × 5 mm, using for photocatalytic activity test. All specimens were cured in water tank (25 °C, RH 100%) for 28 days. However, the photocatalytic board (100 mm × 100 mm × 5 mm) after water curing was then placed in the oven to dry at 105 °C to ensure the oven dry status before photocatalytic test.

### 2.3. Testing

#### 2.3.1. Compressive Strength

To the compressive strength, specimens (40 mm × 40 mm × 40 mm) were put onto the universal pressing machine with the loading rate of 0.3–0.5 MPa until failure. The compressive strength was determined by Equation (1). Each test was conducted three times to obtain the average value.

(1) $$\begin{array}{c} f_{cc}=\frac{F}{A} \end{array}$$

where $f_{cc}$ means the compressive strength of cubic specimens (Mpa), accurate to 0.1 Mpa; F means the failure load (N); and A means the bearing area ($mm^{2}$).

#### 2.3.2. Photocatalytic Characterization

**Figure 3:**
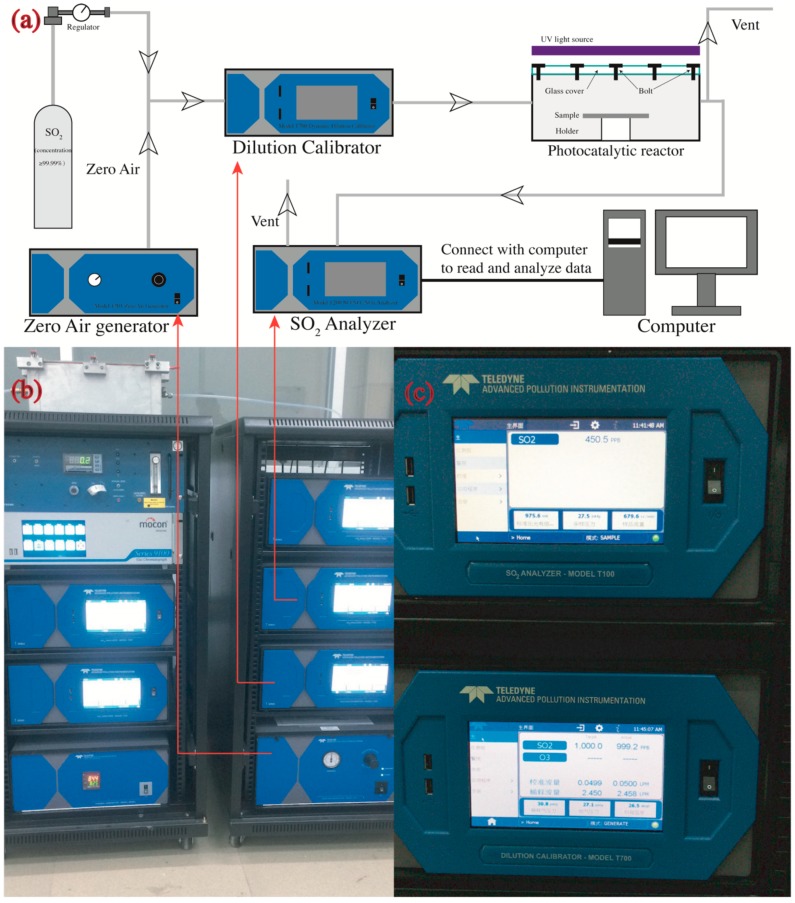
Photocatalytic instruments: (**a**) schematic diagram of measurement geometries (not to scale); (**b**) real picture of photocatalytic instruments; and (**c**) display panel of SO_2_ analyzer (upper) and the dilution calibrator (lower).

The samples prepared by the method in Section 2.2 were placed in a photocatalytic reactor (Figure 1e) to determine their photocatalytic efficiencies. The SO_2_ removal was determined by a continuous flow reactor (see Figure 3) as per a modified code of JIS R1701-1-2010/AMD 1-2011. The reactor is described in detail as follows. The samples were placed in the center of a chamber with internal dimensions of 700 mm × 400 mm × 130 mm. The left side of the chamber was connected to a gas cylinder that provides SO_2_ (purity 99.99%) and a zero-air generator (model 701, Teledyne, Waterloo, Ontario, Canada), while the right side was connected to a SO_2_ analyzer (Model T200, Teledyne, Waterloo, Ontario, Canada) that automatically read the real time concentration of SO_2_ per minute in an accuracy of 1 ppb. Two UV-A fluorescent lamps (2×F8T5 BLB, HRK, South Windsor, CT, USA) were located parallelly onto the chamber to provide ultraviolet light for inducing the photocatalytic reaction. The UV lamps provided a 3 mW/cm^2^ intensity at the peak wavelength of 365 nm measured by a UV light meter (Sentry Optronics Corp., New Taipei City, Taiwan, China).

A complete photocatalytic test mainly contained four steps. First, the SO_2_ was diluted by the zero air to the target initial concentration (~1000 ppb in this study) by a dilution calibrator (Model T700, Teledyne, Waterloo, Ontario, Canada) in an established constant flow rate (3.0 L/min in this study). Second, the diluted SO_2_ flowed through the reactor (with samples) that was covered by a tin foil for about 30 min to fully eliminate existing air in the chamber and to create a full and pure SO_2_ environment in the dark condition (UV lights turn off). This procedure was also helpful to create a gas–solid adsorption–desorption equilibrium in the chamber. Third, the UV lights were turned on under the tin foil and started to irradiate samples, activating the photocatalytic reaction for another 30 min. The temperature and relative humanity in the chamber were recorded by a sensor (SSN-22E, YOWEXA, Shenzhen, Guangdong, China). In this study, the temperature was 25 ± 3 °C, while the relative humidity was 30%. Fourth, the SO_2_ degradation was calculated by Equations (2)–(4).

The physi- and chemisorption (absorption) of SO_2_ (*k*1, %) was calculated as per Equation (2).

(2) $$\begin{array}{c} k1=\frac{{\left[SO_{2}\right]}_{t2}-{\left[SO_{2}\right]}_{t1}}{{\left[SO_{2}\right]}_{t1}}\times 100\% \end{array}$$

where *k*1 is the physi- and chemisorption, ${\left[SO_{2}\right]}_{t2}$ is the final stable concentration of SO_2_ (ppb) at t2 when Step 2 ends, and ${\left[SO_{2}\right]}_{t1}$ is the initial stable concentration of SO_2_ (ppb).

The photocatalytic removal of SO_2_ (*θ*, mg h^−1^ m^−2^) was calculated as per Equation (3).

(3) $$\begin{array}{c} \theta =\frac{\left(\frac{f}{24.45}\right)M\left(SO_{2}\right)\int_{t_{2}}^{t_{3}}\left({\left[SO_{2}\right]}_{\mathrm{inlet}}-{\left[SO_{2}\right]}_{\mathrm{outlet}}\right)\mathrm{dt}}{A\left(t_{3}-t_{2}\right)} \end{array}$$

where *θ* is the amount of SO_2_ removed by the test sample (mg h^−1^ m^−2^), *f* is the flow rate at STP of 25 °C and 1.013 kPa (L min^−1^), M($SO_{2})$ is the molecular weight of $SO_{2}$ (dimensionless), *A* is the surface area of the cement paste samples (m^2^), *t*_2_ is the time to turn on the UV light (h), *t*_3_ is the time to turn off the UV light (h), and 24.45 is the volume of 1 mole of gas at the testing conditions (L). *t*_2_ means the end time of Step 2 while *t*_3_ means the end time of Step 3.

The total degradation (absorption and photo-degradation) of SO_2_ (*k*2, %) was calculated as per Equation (4).

(4) $$\begin{array}{c} k2=\frac{{\left[SO_{2}\right]}_{t3}-{\left[SO_{2}\right]}_{t1}}{{\left[SO_{2}\right]}_{t1}}\times 100\% \end{array}$$

where the *k*2 is the total degradation, ${\left[SO_{2}\right]}_{t3}$ is the final stable concentration of SO_2_ (ppb) at t3 when Step 3 ends, and ${\left[SO_{2}\right]}_{t1}$ is the initial stable concentration of SO_2_ (ppb).

Three replicates of samples were used and each sample was tested at least three times until the standard deviation was less than 5%. The final recorded result is the average of the three plus the standard deviation.

The quantum efficiency was defined as the ratio of the number of molecules undergoing a photocatalytic reaction to the number of quanta (photons) absorbed by photocatalysts, as Equations (5)–(7). The obtained quantum efficiency was 1.25 × 10^−5^.

(5) $$\begin{array}{c} \phi =\frac{N_{molecule}\;\left(mol/s\right)}{N_{photon}\;\left(mol/s\right)} \end{array}$$

(6) $$\begin{array}{c} N_{molecule}\;\left(mol/s\right)=\frac{f\times \left(SO_{2,in}-SO_{2,out}\right)}{24.45} \end{array}$$

(7) $$\begin{array}{c} N_{photon}\;\left(mol/s\right)=\frac{IA}{N_{A}hv}=\frac{IA\lambda t}{N_{A}hc} \end{array}$$

where *f* is the flow rate, 3 L/min = 5 × 10^−2^ L/s; 24.45 is the volume (L) of 1 mol gas in the test condition; $SO_{2,in}\;and\;SO_{2,out}$ are respectively real-time the inlet and outlet concentration of SO_2_, ppb (1 ppb = 10^−9^); $N_{A}$ is the Avogadro constant, 6.02 × 10^23^; *I* is irradiation of UV light (365 nm), 3 mW/cm^2^ = 30 W/m^2^ = 30 J/(s·m^2^); *A* is the area suffered the irradiation, 100 mm × 100 mm = 0.01 m^2^; *h* is the Planck constant, 6.62607015×10^−34^ J·s; *v* is the optical frequency, c (light speed)/λ(wavelength), s^−1^; *c* is the light speed, 3 × 10^8^ m/s; *λ* is the wavelength, 365 nm = 3.65×10^−7^ m; and *t* is the time, 1 s.

#### 2.3.3. Response Surface Methodology

The response surface methodology (RSM) is a method that uses multiple quadratic regression equation to fit the functional relationship between factors and response values and seeks the optimal process parameters by analyzing the regression equation. Traditional experimental design and optimization methods fail to give intuitive graphics, and therefore cannot observe the optimum points intuitively. Although the optimum values can be found, it is difficult to identify the optimum regions intuitively. Actually, a conventional methodology of one factor at a time (OFAT), which means a single factor for a specific experimental design with other factors maintained constant, has been blamed for failing to provide expected output as the interaction effects among variables are not correctly examined [21]. On the contrary, RSA as an optimization method is created to undertake the central composite design (CCD). It creates a function of systematic response as one or more factors. Afterwards, graphic technique is used to display the function relationship, allowing researchers to easily select the optimum conditions in experimental design by intuitive observation. Theoretically, it is a key statistical method for solving multivariate problems. RSM is widely used in research to identify the optimum solution by analyzing variables which affect final results [21,22,23,24,25,26].

Here, RSM was applied to analyze how oxygen content and humidity influence the SO_2_ removal jointly. Table 4 displays a summary of number of specimen and data retrieved for each mortar. In particular, the oxygen content at given ppb was released and controlled by oxygen gas cylinder coupled with dilution calibrator, similar to sulfur dioxides demonstrated in Section 2.3.2. On the contrary, the humidity was applied and regulated by the number of water spays realized by a professional sprinkler. In particular, each press of the sprinkler can spread 5 mL water onto the surface of mortar. The mortar notation M5-x means mortar M5 shown in Table 3 plus x number of water spays. For example, M5-2 indicates the mortar which used 100% NT@RFA as fine aggregates and suffered two sprays.

Above of all, a mathematic model relying on the multivariate linear regression with the second order was established as Equation (8).

(8) $$\begin{array}{c} y=\beta_{0}+\beta_{1}x_{1}+\beta_{2}x_{2}+\beta_{3}x_{1}x_{2}+\beta_{4}x_{1}^{2}+\beta_{5}x_{2}^{2}+\varepsilon \end{array}$$

where $\beta_{i}\;\left(i=0,1,2,3,4,5\right)$ is the coefficient, $x_{1}\;and\;x_{2}$ are variables, and ε is the total error.

The coefficients, namely $\beta_{i}$, could be solved by the least square method (LSM) based on the established significance threshold level of 0.05, namely α=0.05. Since the values of selected variables, namely oxygen content and humidity, were of different orders of magnitude and of different units, the values ertr pretreated by data normalization into dimensionless values between 0 and 1 according to Equation (9).

(9) $$\begin{array}{c} x^{\prime }=\frac{x-\min\left(x\right)}{\max\left(x\right)-\min\left(x\right)} \end{array}$$

where $x^{\prime }$ indicates to the normalized x, while max(*x*) and min(*x*) represent the maximum value and the minimum value of all *x*, respectively.

Before regression analysis, analysis of variance (ANOVA) was used to test the significance of mean difference between two or more samples. Due to various factors, the data obtained fluctuated. The causes of fluctuations can be divided into two categories, namely uncontrollable random factors and the controllable factors imposed by the study. ANOVA starts with the variance of the observed variables and aims to identify target variables that generate significant influence on the observed variables. It provides a qualitative relationship of the influence degree of each variable on the results, so as to eliminate the variables that have less influence on the results and to improve the efficiency and accuracy of the experiment. On the contrary, regression analysis was used to study the quantitative relationship between variables and results, finding the corresponding mathematical model. In the regression analysis, it was necessary to analyze the variance of the influence of each variable on the results, so as to eliminate the variables that have little influence and to improve the effectiveness of the regression analysis. Overall, variance analysis, no matter how complex the relationship between variables (factors and results) is, can always get the overall judgment of whether the factors have a significant impact on results. Only those factors with significant impact are left for regression analysis.

The achieved regression model and coefficients were then tested for significance according to p-value (t test for coefficients and F-test for the overall regression) and the R square (R^2^). In particular, when the LSM is used in statistics to estimate the parameters in linear regression analysis, R^2^ is the ratio of the sum of regression squares to the sum of total deviation squares, which indicates the proportion of the sum of total deviation squares that can be explained by regression squares, namely the goodness of fit of regression curve. The larger is the proportion, the better is the model and the more accurate is the regression effect. R^2^ is between 0 and 1, and the closer it is to 1, the better the regression fitting effect is. Generally speaking, a goodness of fit of the model over 0.8 is accepted [27].

After calculating and examining the model, the mathematical model of the response to each factor level can be obtained, and the relationship between the response and the factors can be plotted graphically. The three-dimensional surface (i.e., a two-factor response surface) is constructed by taking the two-factor level as X coordinate and Y coordinate and the corresponding response calculated from the formula as Z coordinate. The above procedures were conducted by Matlab_R2015b (MathWorks, Natick, MA, USA) and StataSE_13 software (StataCorp, College Station, Texas, USA).

#### 2.3.4. Environmental Scanning Electron Microscope (ESEM)

Environmental scanning electron microscopy (ESEM, Quanta FEG 250, FEI, Hillsboro, OR, USA) was used to observe the morphologies and microstructures of the CPs. Samples were placed directly under the lens to obtain the micrographs. Elemental micro-spectroscopy was performed by energy-dispersive X-ray spectroscopy (EDS, Oxford Instruments, Scotts Valley, CA, USA).

#### 2.3.5. Microstructure Analysis

The changes in the mortar microstructure before and after SO_2_ degradation were determined by mercury intrusion porosimetry (MIP, Poresizer 9320, Micromeritics, Norcross, Georgia, USA) with a maximum intrusion pressure of 210 MPa. A contact angle of 140° and a cylindrical pore geometry were assumed.

#### 2.3.6. Weathering Test

Weathering tests were performed to test the durability and reuse capacity of the prepared mortar samples. Considering the actual use situation, two main weathering conditions of raining and abrasion were simulated by water washing and sandpaper grinding, respectively. One cycle of water washing (about 1 min) and oven drying was defined as one reutilization. To ensure the same humidity level, all specimens were dried in an oven at 100 ± 5 °C for 12 h after washing. To simulate natural abrasion caused by activities such as wind, human touching, and stepping [28], one abrasive paper of grade CW 220-2c was utilized to abrade the sample surface by sweeping back and forth 500 times. To ensure equivalent abrasion conditions (friction forces) for all samples, force applied perpendicular to the direction of gravity and the friction coefficient determined by the surface friction was maintained as a constant. The former was realized by attaching the abrasive paper to the surface of a brick with a constant weight; the latter was achieved by using a new abrasive paper for each testing sample.

## 3. Results and Discussion

### 3.1. Effect of NT@RFA Content on Mechanical Strength and Total SO_2_ Degradation of Photocatalytic Mortar

**Figure 4:**
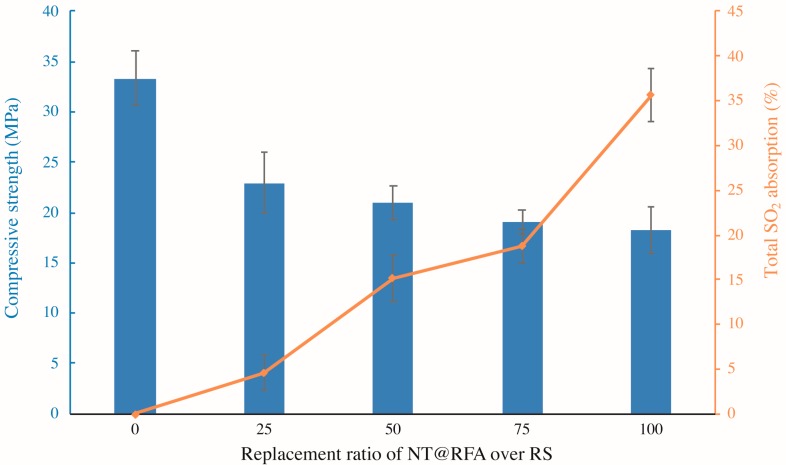
Compressive strengths (28 days) and total SO_2_ degradation of photocatalytic mortars containing different proportions of NT@RFA.

Figure 4 illustrates the compressive strength and total SO_2_ degradation of the photocatalytic mortars with different replacement ratios of NT@RFA for RS. As shown, increases in the replacement ratio induce decreases in the compressive strength. This is because the crushing indices of RFA is lower relative to that of RS [29]. Thus, the use of NT@RFA deteriorates the mechanical performance of the photocatalytic mortar. On the contrary, NT@RFA content is positively related to the total SO_2_ degradation. It is because the NT@RFA functions as photocatalyst and more photocatalysts indicate a better photocatalytic performance. In particular, the increase of total SO_2_ degradation from the replacement ratio of 75% to 100% is the most significant. A possible reason was the overlapping effect. That is, when the content of NT@RFA is less than 75%, some photocatalysts are likely to be covered by RS or hydration products of cement, leading the effective quantity of photocatalysts on the mortar surface declined. Particularly, it is reckoned that the photocatalytic efficiency declines once NT@RFA is covered by RS because the content of NT on RS is 0% (0 over 0.0023 g in Table 2) relative to NT@RFA. However, once the NT@RFA content reaches 100%, even though some NT@RFA are still covered, the mortar surface is all filled with NT@RFA, maximizing the photocatalysis.

### 3.2. Effect of Different CPs on the SO_2_ Removal

**Figure 5:**
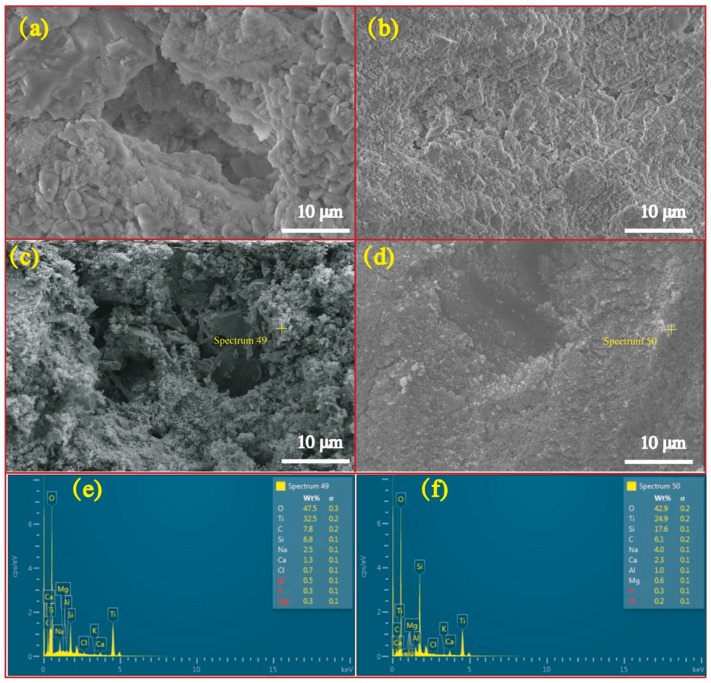
Micrograph and EDS profiles: (**a**) micrograph of pure FRA; (**b**) micrograph of pure RS; (**c**) micrograph of NT@FRA; (**d**) micrograph of NT@RS; (**e**) EDS profile of NT@FRA at selected point; and (**f**) EDS profile of NT@RS at selected point.

Figure 5 exhibits micrographs and correlated EDS profiles of RFA and RS before and after loading NT. As is illustrated, the FRA is featured by the tough surface and high porosity while the RS has a higher level of smoothness relative to FRA. This feature determines FRA’s higher capacity to load more NT onto its surface and into pores. Actually, to the prepared NT@RFA, some particles are present on the RFA surface. EDS observation showed that these particles are NT with high concentrations of Ti, which is not abundant in RFA, as proved by Table 1. These data verify that the soaking method successfully deposited NT photocatalysts on the RFA base material. As a contrast, NT@RS holds few NT particles on its surface. This result obtained by the micro-measurement echoes the previous macroscopic result shown in Table 2, namely the NT loaded by per gram of RFA and RS are, respectively, 0.0023 and 0.0015 g.

**Figure 6:**
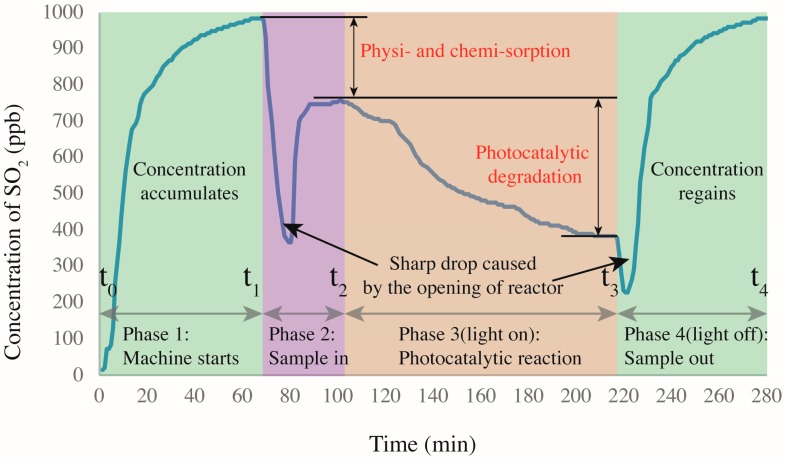
SO_2_ degradation in presence of CP-incorporated specimen: the whole degradation process.

Figure 6 shows the SO_2_ degradation process as four main sections. The first section begins at zero concentration when the reactor gas flow starts and ends at *t*_1_ when the SO_2_ concentration reaches the target of ~1000 ppb with a stability of <1 (0.65 at *t*_1_). The initial concentration is not always 1000 ppb because of various complex factors that are difficult to control accurately, such as sealing. However, the fluctuation is limited to ±20 ppb for each test. In the second section (samples are placed in the reactor), the concentration decreases significantly because the cover of the reactor is opened to place the specimens. After the cover is replaced, the concentration re-equilibrates at a peak value somewhat below that at *t*_1_. Because the UV light is not turned on, the decrease in concentration is obviously not caused by photocatalytic reaction. By referring to [17], the decrease is ascribed to the physi- and chemisorption of the acidic SO_2_ by the alkaline silicates of the mortar, as in carbonation. Hydration products such as calcium hydroxides induce sample alkalinity [30], which generates a neutralization effect. This effect was also proved by Chen et al. [15]. In addition, the porous structure and pore water allow some physisorption because SO_2_ can react with H_2_O under near-ambient conditions [31]. At *t*_2_, the UV light is turned on, marking the beginning of the photocatalytic reaction process. Naturally, the concentration of SO_2_ is continuously decreased with increasing time before reaching a plateau. When activated by the UV light, the NT loaded on the CP surface generates photo-activated electrons and holes [32]. These electron–hole pairs then participate in redox reactions with ambient O_2_ and H_2_O molecules, generating oxidants that further oxidize SO_2_ into SO_4_^2−^ radicals, which are less harmful and can be washed away by water. Another concentration decrease occurs after *t*_3_ because the reactor is again opened to remove the sample. Eventually, the SO_2_ concentration recovers to ~1000 ppb.

**Figure 7:**
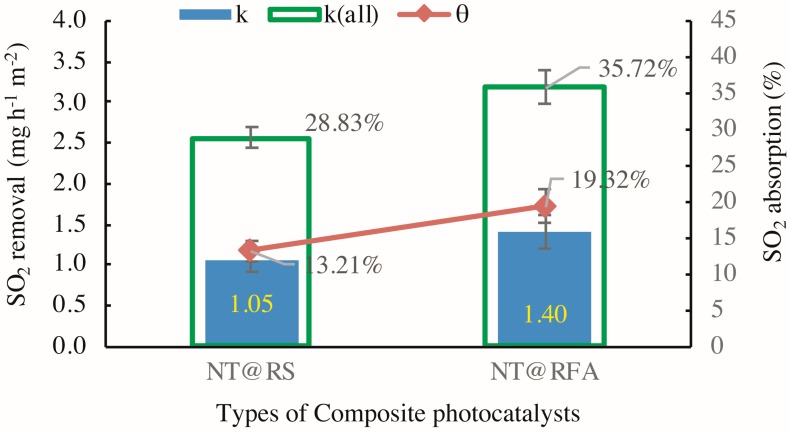
SO_2_ degradation in presence of CP-incorporated specimen: the detected quantitative SO_2_ removal of samples containing different CPs (*k* means *k*1 in Equation (2) and *k*(all) means *k*2 in Equation (4)).

Figure 7 exhibits the varied performances of different CPs in the experimental cycles identified by Figure 5. The SO_2_ removals *k* for NT@RS (M1 in Table 3) and NT@RFA (M5 in Table 3) are 1.05 and 1.40 mg h^−1^ m^−2^, respectively. The increasing trend follows the photocatalytic activity scaling with porosity [13]. The highly porous RFA holds more hydrated products and more final reaction products while providing more active sites for photocatalysis [20]; therefore, RFA shows a higher removal. Regarding to SO_2_ absorption, the sequence maintains the same. A higher absorption is achieved by NT@RFA because it has a higher total alkalinity. As stated above, the chemisorption generated by the neutralization effect between the alkaline silicates and the acidic SO_2_ dominates the adsorption. The RFA is characterized by a high proportion of old mortar attached [33,34]. This old mortar contains abundant calcium hydroxides produced by hydration. This is the reason NT@RFA shows a higher absorption, which is ~46% higher than NT@RS. This enhancement of 46% is higher than the enhancement (~33%) of NT@RFA over NT@RS in regard to *k*. It shows that the NT@RFA is more characterized by high alkalinity rather than high porosity relative to NT@RS.

Previous researchers mainly attempted to mix various elements with NT to enhance the photo-degradation efficiency [35,36,37]. However, element admixture increases the cost and is impractical for large-scale application in the construction industry. In addition, while many studies investigated the degradation of SO_2_, they mainly used only NT as a photocatalyst, or NT coated on limestone [38], Teflon plates, glass fiber filters, nanofibers [39], or glass beads [40,41]. These yielded catalysts totally unlike building materials, such as mortar containing intermixed high-porosity alkaline CPs, as in this research. More significantly, previously published works have used varied experimental conditions and are thus incomparable with each other, impeding cross-comparison of reported data. For instance, it was observed that 4000 ppm SO_2_ could be degraded by NT powder under 365-nm radiation at 5.3 mW/cm^2^ [42]. However, lower-intensity (0.75 mW/cm^2^) radiation at a similar wavelength failed to degrade 0.2 ppm SO_2_ on NT-coated glass fiber and Teflon plates [43]. Considering the various experimental parameters applied in individual studies, the systematic experimental research undertaken in this study is significant to design building materials intermixed with RFA-based CPs.

### 3.3. Integrated Effect of Oxygen Content and Humidity on the SO_2_ Removal

**Figure 8:**
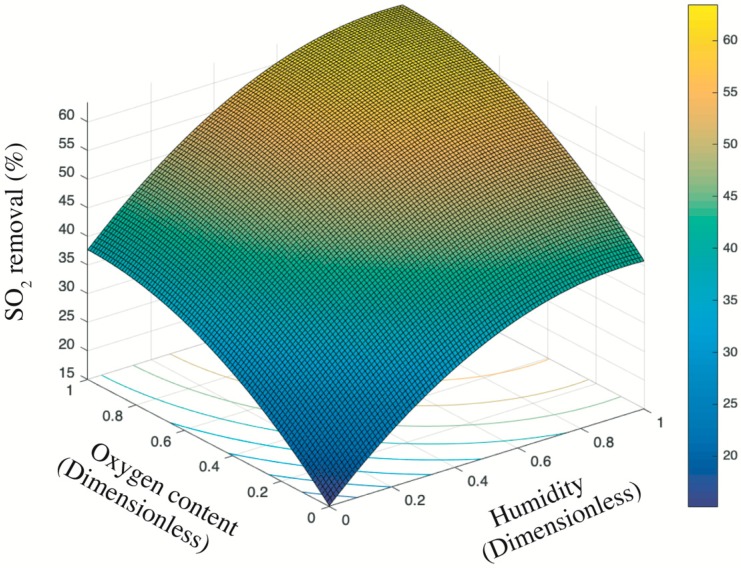
Trend surface of sulfur dioxides removal.

**Figure 9:**
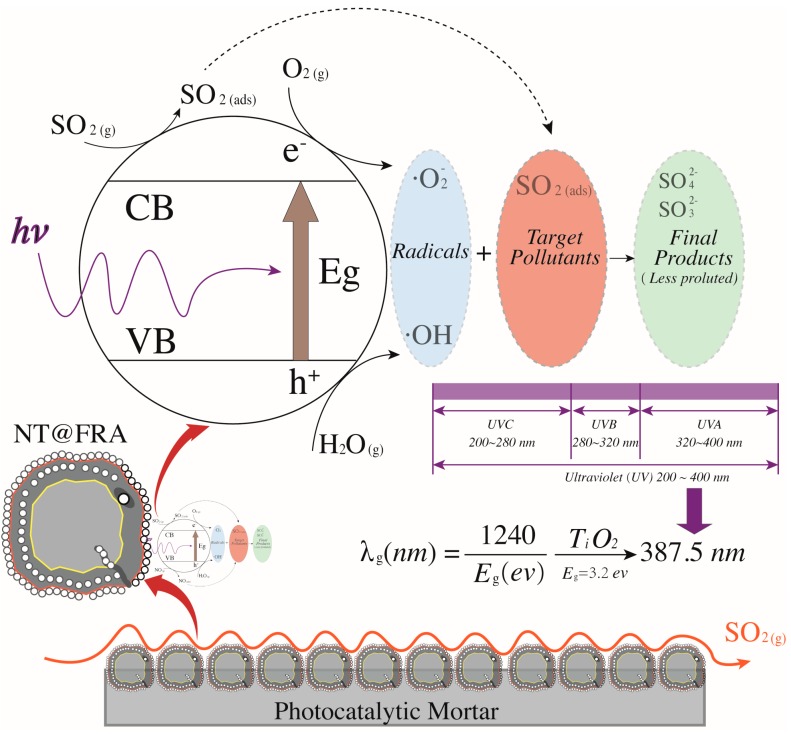
The reaction mechanism of photocatalytic oxidation of sulfur dioxides.

Figure 8 presents the trend surface of sulfur dioxides removal as a function of the oxygen content and humidity. In particular, considering the different order of magnitude and units of measurement between oxygen content and humidity (e.g., 200 ppb vs. 2 times of sprayings), all variable data were first normalized into 0–1 basing on Equation (5). Thus, both the x-axis and y-axis are dimensionless values between 0 and 1. Based on the color bar located to the right side of Figure 8, the brighter part is of relatively higher sulfur removal in comparison to the darker place. Thereby, both the humidity and the oxygen content are positive variables that contribute a higher photocatalysis. It is because the superoxide, one of the radicals degrading pollutants (see Figure 9), is generated by the reaction between oxygen molecules and photogenerated electrons [35,44]. The enhancing of oxygen level accelerates the oxygen–electron reaction and produces more superoxide. Besides, the formed superoxide is beneficial on consuming electrons, while this electron-scavenging feature is conducive to the separation of electron–hole pairs [35,45]. On the contrary, the water molecules are responsible for reacting with photogenerated holes to form the hydroxide group, which is another vital radical in photocatalysis (see Figure 9). These hydroxide groups are both absorption sites for sulfur dioxides and can catch more water molecules by hydrogen bonding, resulting in more hydroxides [46,47]. Furthermore, the water molecules can function as self-clearing agents to delay or ease the deactivation of photocatalysts. It is because the deposited sulfite, a typical final product generated during the photocatalytic reaction, can load on the surface of photocatalysts. It to some degree blocks the active sites and holds back the upcoming sulfur dioxides conversion, leading to the deactivation [43]. However, in the presence of water molecules at certain quantities, those sulfite or sulfate previously loaded onto the surface of photocatalysts might be adsorbed and converted into water-dissolved products that can be taken away from the surface by gas [48].

**Figure 10:**
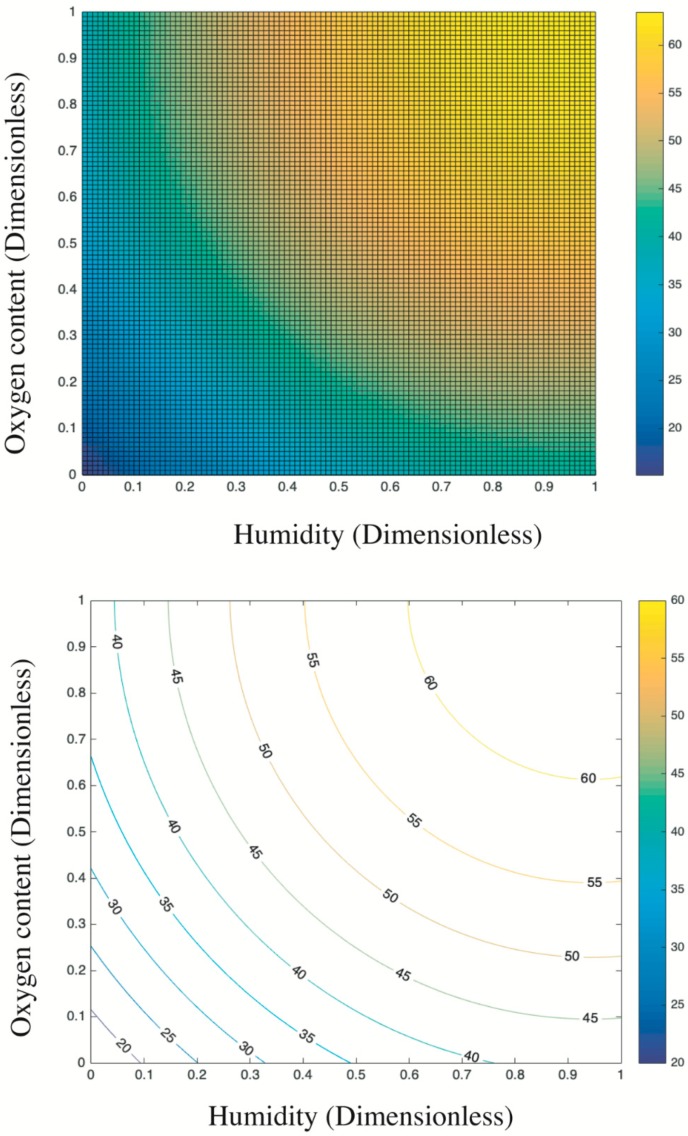
Contour plots of estimations: top view of the trend surface (**upper**); and the quantitative contour plot (**lower**).

Based on the above analysis, it is still unclear which variable plays a decisive role. Then, the contour plot in Figure 10 illustrates horizontals curve formed by projecting points of the same height on the surface into a loop directly onto the plane. That is, varied combinations of oxygen content and humidity can lead to the same photocatalysis, such as (0.2, 0) and (0, 0.25), or (0.4, 0.8) and (0.13, 0.4). Remarkably, the marginal contribution of each added humidity is higher than that of oxygen content. In particular, the maximized humidity can lead to 45% sulfur removal while the oxygen content to its top limit fails to achieve the same photocatalysis. Therefore, the humidity relative to oxygen content is a more evident and significant contributor upon photocatalysis. This result is consistent with previous research [17] that states the radicals generated by water in comparison to radicals induced by oxygen have a 24 times faster reaction rate. Besides, the oxidation potential of hydroxyl radical (2.8 V) is about 60% higher than hydrogen peroxides (1.78 V), leading the former the dominant oxidant [49]. That is, hydroxyl radicals are more crucial oxidants relative to superoxide and thus are more reliable to oxidize sulfur dioxides.

Overall, the above analysis provides an economic approach to boost photocatalysis by increasing the humidity in practice because the water is much cheaper than oxygen at least in this experiment. For example, the target photocatalysis of 55% can be achieved either by 0.4 humidity plus 0.9 oxygen content or by 1 humidity plus 0.42 oxygen content. Even though some research highlights that an over-level humidity might block the light irradiation and lead to a negative effect upon photocatalysis [49], this phenomenon is not detected by this experiment considering the established data limit.

At the end of this section, the ANOVA results and the modeled regression equation are displayed. Actually, these two parts should be addressed before trend surface and contour plot analysis since the response surface is established based on the regression results. However, considering the level of significance in regard to this research, the ANOVA plus regression model are presented in brief here. 

Based on the ANOVA results presented by Table S1 (available in the Supplementary Materials), all linear variables (*x* and *y*) and squares (*x*^2^ and *y*^2^) are significant due to the low p-value considering the given significance threshold of 0.05. However, the interaction effect presented by *xy* is totally insignificant. By removing this effect, all other four factors of Equation (4), namely *x, y, x*^2^ and *y*^2^, are significant, as shown by the right sub-table of Table S1 and should be taken into consideration of multiple linear regression. 

By Table S2 (available in the Supplementary Materials) and Equation (4), the regression model is written as Equation (10),

(10) $$z=15.16+54.73x+44.60y-29.02x^{2}-22.10y^{2}$$

where the *z* (%) indicates to the total degradation of sulfur dioxides, *x* indicates to the oxygen content (ppb), and *y* indicates to the humidity (num. of spraying). In particular, both the entire equation and the specific coefficients are significant because all p-values are less than 0.05 while the R-square is 0.98, which is higher than 0.8 [27].

### 3.4. Reutilization and Durability of CPs

The regeneration capacity of photocatalysts is embodied by the reusability in this study. For photocatalysts, the reusability is critical because it determines the real use cost in practical application. Greater photocatalyst reusability corresponds to a lower overall cost and thus greater potential applicability. Therefore, reutilization must be characterized. 

After one use, the CPs retain some of the final products, which occupy some of the active sites expected to participate in photocatalysis. The effect of this occupation may be initially negligible, but over time, would gradually decrease the photocatalytic efficiency. As observed by Vorontsov et al. [50], both monodentate and bidentate ligands were available for the sulfate species upon the NT surfaces. Unlike the firmly combined bidentate species, the monodentate species were relatively easily removed by washing. The passivation effect generated by bidentate sulfate was permanent and could not be removed only by physical methods. However, if the chemical method were used to remove them, then a more in depth and complicated study would need to be conducted, which is not the scope of this study.

**Figure 11:**
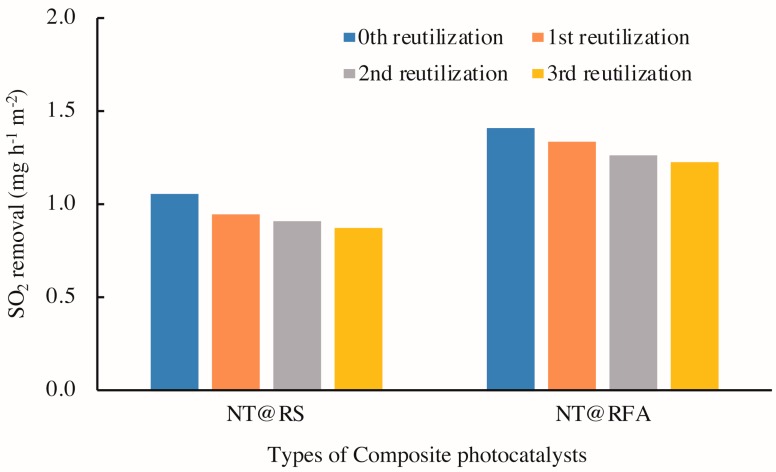
SO_2_ degradations of different samples: reused 0–3 times.

Figure 11 illustrates the photocatalytic activities of CPs after 0–3 cycles of washing with deionized water. The results show a clear decrease in photocatalytic activity with increased reutilization, i.e. washing times, for all specimens. This is also supported by the changed porosities of the samples. As detected by mercury porosimetry, the porosities of NT@RFA before and after SO_2_ degradation were 24.56% and 18.76%, respectively. A positive correlation between porosity and photocatalytic activity was also reported by Poon & Cheung [51]. However, for specific composites, the amount of decrease of NT@RFA is 6.1% less than NT@RS. This phenomenon is partly attributed to the permanent generation of bidentate sulfate species that cannot be washed away by water. As recorded by the authors of [40,50], the accumulation of bidentate sulfate ligands permanently deactivates the catalysts. In addition, the bonding force between NT and carriers also affected the reduction [52]. Almost all NT particles on the carrier surfaces of RFA and RS are attached via physical adsorption, which is closely related to the carrier surface characteristics. This is also verified by the testing results (see Table 2) of the amount of NT attached to and adsorbed by the various carriers. The RFA shows a stronger bonding force; its rough surface and high porosity are directly reflected by Figure 5. This strong bonding limits and overcomes the releasing force provided by the water stream during washing, thereby retaining the relative larger amount of surface-loaded photocatalysts. This explains the highly stable performance of NT@RFA after two use cycles. It also implies that NT@RFA shows better long-term durable performance, because rain is a critical weathering factor experienced by building materials in practical application.

**Figure 12:**
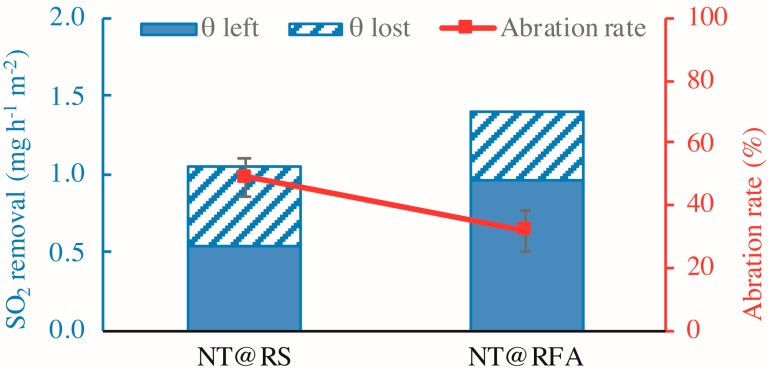
SO_2_ degradations of different samples: before and after abrasion.

The durability is mainly embodied by the abrasion resistance of the mortar, as determined by the retained photocatalytic activity after mechanical abrasion. Figure 12 shows the SO_2_ removal rates of all samples before and after abrasion. The figure reveals a significant loss (>25%) in photocatalytic efficiency for all samples after abrasion. This is caused by the disruption of physical bonding between photocatalysts and carriers, as stated previously. Similarly, the level of reduction of NT@RFA is lower than NT@RS. Other than the rough surface of RFA, NT adsorbed into deep pores also contributes to its performance retention. The low strength of RFA facilitated abrasion, but after abrasion, the photocatalysts contained in the deep pores are exposed to UV irradiation. This somewhat compensates for the photocatalytic loss induced by abrasive removal of surface NT.

## 4. Conclusions

Based on the above demonstrations and analyses, the following conclusions could be drawn.

(1) The prepared CPs increased the use value of C&DW because they endowed the waste materials with photocatalytic capability. In addition, the CPs facilitated the dispersion of NT within cementitious materials. This avoided agglomeration and provided a relatively larger surface area for effective UV irradiation.
(2) The SO_2_ degradation comprised two processes of physiochemical absorption followed by photocatalytic removal, because the acidic SO_2_ could react with the alkaline new mortar and old mortar attached to RFA.
(3) The sample containing NT@RFA achieved ~46% higher adsorption and ~24% higher total degradation than that with NT@RS because of its advantageous high alkalinity and high porosity.
(4) Both reutilization and abrasion decreased the SO_2_ degradation capacities of all CPs. However, NT@RFA, because of its high porosity, showed relatively higher tolerance to weathering.
(5) The final product, viz. the photocatalytic mortar, can be used in any place in the building, such as the wall, floor, or even roof.

The prepared mortar contains some sulfuric acid and should be carefully used. However, the mortar we prepared is still safe due to the following aspects. First, the mortar is used to degrade sulfur dioxide existing in atmosphere. The concentration is quite low and most of the time is less than 1 ppm. This low concentration correspondingly leads to low content of final sulfuric acid held by the mortar. Only few milligrams per square meter were obtained by the research result in this study. Second, the mortar is expected to suffer washing either by natural rains or by artificial washing. During the occasional washing process, some sulfuric acid can be removed. Definitely, the water containing sulfuric acid should be carefully re-collected by special channels and then degraded into pollution-free water before final discharging. To the end, the neutralization of mortar before the disposal is significant. Furthermore, the regular maintenance such as washing as stated above is also important since the sulfur dioxide degradation is a continuous process taken place during the entire service life of the building.

## Figures and Tables

**Table 1:** Chemical compositions and physical properties of materials.

Materials	SiO_2_	Fe_2_O_3_	Al_2_O_3_	CaO	MgO	SO_3_	LoI *	Density	Water Absorption	Size
	(%)	(%)	(%)	(%)	(%)	(%)	(%)	(kg/m^3^)	(%)	(mm)
Cement	19.61	3.32	7.33	63.15	2.54	2.13	1.92	3160	-	-
RFA	59.63	4.66	16.3	14.33	1.5	1.43	2.15	2387	6.8	1.18–2.36
RS	96.18	0.06	2.76	-	-	-	1.00	2651	0.85	1.18–2.36

* Loss on ignition.

**Table 2:** Mixture proportions of different CPs.

Mix. Notation	RFA (g)	RS (g)	NT (P25) (g)	Water (mL)	P25 Absorption (g)
NT@RFA	80	-	1.0	100	0.0023 (per gram of RFA)
NT@RS	-	80	1.0	100	0.0015 (per gram of RS)

Note: The P25 absorption was determined by an analytical balance with accuracy of 0.0001 g.

**Table 3:** Mix proportion of photocatalytic mortar.

	Cement	Water	NT@RFA	NT@RS	Replacement Ratio of NT@RFA over NT@RS (%)
M1	1	0.5	-	2.5	0
M2			0.625	1.875	25
M3			1.25	1.25	50
M4			1.875	0.625	75
M5			2.5	0	100

**Table 4:** Experimental design.

Mortar Notation	Oxygen Content (ppb)						Test
	0	200	400	600	800	1000	SO_2_ Removal
M5-0	3	3	3	3	3	3	18
M5-1	3	3	3	3	3	3	18
M5-2	3	3	3	3	3	3	18
M5-3	3	3	3	3	3	3	18
M5-4	3	3	3	3	3	3	18
M5-5	3	3	3	3	3	3	18
Total	18	18	18	18	18	18	108

## Supplementary Materials

The following are available online at https://www.mdpi.com/2079-4991/9/11/1533/s1, Table S1: ANOVA analysis, Table S2: Multiple linear regression analysis.
