# Supplementary material for: Sulfur Dioxide Degradation by Composite Photocatalysts Prepared by Recycled Fine Aggregates and Nanoscale Titanium Dioxide
Source: Nanomaterials (Basel). 2019 Oct 29;9(11):1533. doi: 10.3390/nano9111533 (PMC6915600; doi:10.3390/nano9111533)
Supplement: Supplementary file 1 [file nanomaterials-09-01533-s001.pdf]

Supplementary materials

Table S1. ANOVA analysis

Number of obs = 36, R-squared = 0.9771
Root MSE = 2.04983, Adj R-squared = 0.9733

Source	Partial SS	df	MS	F	Prob > F
Model	5380.19641	5	1076.03928	256.09	0.0000
x	846.580794	1	846.580794	201.48	0.0000
y	562.106294	1	562.106294	133.78	0.0000
xy	4.4670e-14	1	4.4670e-14	0.00	1.0000
x2	301.785676	1	301.785676	71.82	0.0000
y2	175.017833	1	175.017833	41.65	0.0000
Residual	126.053593	30	4.20178644		
Total	5506.25	35	157.321429		

Number of obs = 36, R-squared = 0.9771
Root MSE = 2.01649, Adj R-squared = 0.9742

Source	Partial SS	df	MS	F	Prob > F
Model	5380.19641	4	1345.0491	330.78	0.0000
x	989.212863	1	989.212863	243.27	0.0000
y	656.810054	1	656.810054	161.53	0.0000
x2	301.785676	1	301.785676	74.22	0.0000
y2	175.017833	1	175.017833	43.04	0.0000
Residual	126.053593	31	4.06624495		
Total	5506.25	35	157.321429		

Note: *x* indicates the variable of humidity, while *y* refers to the oxygen content.

Table S2. Multiple linear regression analysis.

Source	SS	df	MS
Model	5380.19641	4	1345.0491
Residual	126.053593	31	4.06624495
Total	5506.25	35	157.321429

Number of obs =	36
F(4, 31) =	330.78
Prob > F =	0.0000
R-squared =	0.9771
Adj R-squared =	0.9742
Root MSE =	2.0165

z	Coef.	Std. Err.	t	P>|t|	[95% Conf.	Interval]
x	54.73214	3.509088	15.60	0.000	47.57531	61.88897
y	44.59821	3.509088	12.71	0.000	37.44138	51.75504
x2	-29.01785	3.368315	-8.61	0.000	-35.88758	-22.14813
y2	-22.09821	3.368315	-6.56	0.000	-28.96794	-15.22849
_cons	15.16071	1.000213	15.16	0.000	13.12077	17.20066
